# Echocardiographic Assessment of Left and Right Ventricular Systolic Function Beyond Ejection Fraction

**DOI:** 10.3390/life16101731

**Published:** 2026-10-15

**Authors:** Nikoleta Hadjigeorgiou, Tamara Stosic, Despo Melanarkiti, Maria Ioannou, Alexandros Patrianakos, Angeliki Mouzarou

**Affiliations:** 1 Department of Cardiology, Limassol General Hospital, State Health Services Organization, 4131 Limassol, Cyprus; 2 Department of Cardiology, Heraklion University General Hospital, 71110 Heraklion, Crete, Greece; 3 Department of Cardiology, Paphos General Hospital, State Health Services Organization, 8026 Paphos, Cyprus

**Keywords:** left ventricular systolic function, right ventricular systolic function, echocardiography, ejection fraction, ventriculo–arterial coupling, 3D echocardiography, speckle-tracking echocardiography, myocardial work, cardiac power output

## Abstract

The assessment of left and right ventricular systolic function is vital information for the management of cardiac disease. Everyday clinical practice has traditionally relied on the measurement of the ejection fraction (EF). Nonetheless, EF has known limitations and may not always accurately reflect myocardial systolic performance. Intrinsic myocardial contractility may decline long before reductions in left or right ventricular EF become apparent. Adjunct parameters such as tissue Doppler-derived systolic velocities of the mitral and tricuspid annulus may detect such early abnormalities; however, they do not represent global systolic performance. Consequently, commonly used indices may exhibit suboptimal performance for the detection of systolic dysfunction. Given the limitations of current measurement methods, there is growing concern about the reliability of heart failure (HF) classification based solely on EF. The integration of advanced echocardiographic indices, such as speckle-tracking-derived strain, three-dimensional (3D) volumetric assessment, myocardial work (MW), and cardiac power output (CPO), in combination with classical parameters provides more accurate, reproducible, and clinically meaningful diagnostic and prognostic information in patients with HF. Nevertheless, despite their potential, these techniques remain underutilized in routine practice. Early identification of systolic dysfunction and timely initiation of therapy can substantially improve clinical outcomes. In this setting, this review aims to comprehensively examine both established and emerging echocardiographic techniques used for the assessment of ventricular systolic performance, with the objective of promoting their effective integration into routine clinical practice.

## 1. Introduction

The assessment of ventricular systolic function, particularly of the left ventricle, has long been a subject of debate within the scientific community [1,2,3,4]. This reflects the limitations of conventional imaging techniques in capturing complex myocardial mechanics. Accurate evaluation is essential, as ventricular dysfunction has major therapeutic and prognostic implications across a wide range of cardiovascular diseases.

From an anatomical, physiological, and embryological perspective, the left and right ventricle share several similarities but also important differences [5]. In anatomically normal hearts, right ventricular (RV) and left ventricular (LV) systolic functions mirror each other. Differences in the appearance of RV size on traditional two-dimensional (2D) imaging result from the anatomic location of the right ventricle in the thoracic cavity and the inability of a planar 2D ultrasound beam to capture a 3D structure. Although the right ventricle appears smaller than the left ventricle in most 2D projections, in normal individuals the total RV volume is actually larger [6]. The ventricles differ in their architecture, as despite both being composed of the same myocardial fibers, these fibers are differently orientated, consequently, leading to distinct contraction patterns [4,7]. In both ventricles, contractile function depends on both heterometric and homeometric mechanisms, which are fundamentally similar. However, they respond differently to changes in loading conditions [8,9]. The RV tolerates volume overload more effectively but is more sensitive to increases in afterload [5]. Moreover, RV contraction is strongly influenced by interventricular septal function, reflecting the principle of ventricular interdependence [10]. Overall, the shared features and distinct characteristics of the left and right ventricle highlight the importance of a tailored approach in the assessment of systolic function.

Despite advances in diagnostic techniques, one of the most frequent challenges in clinical practice remains the accurate quantification of both LV and RV systolic performance [11,12]. This is of major clinical importance, as it directly affects therapeutic decisions and has prognostic implications. Echocardiography represents an accessible, cost-effective, repeatable, and widely available modality to evaluate cardiac structure and function [13]. For the assessment of LV and RV systolic function, several conventional echocardiographic indices (such as EF) have been proposed [11,14,15]. Left ventricular ejection fraction (LVEF), despite its caveats, continues to be widely used in daily clinical practice [11]. In addition, newer echocardiographic parameters, (such as speckle-tracking-derived strain) may provide earlier detection of systolic dysfunction. Other modalities, including cardiac magnetic resonance (CMR), remain the gold standard for quantitative assessment of biventricular volumes and function [3,16]. In clinical practice, echocardiography remains the first-line modality for imaging the left ventricle and right ventricle, while advanced technologies can provide complementary information [11,12,15].

The purpose of this review is to emphasize the importance of accurate assessment of left and right ventricular systolic function, which is not adequately reflected by EF alone, and to discuss the clinical applicability of advanced echocardiographic indices in daily practice. Unlike previous reviews that have primarily focused on individual imaging parameters or single ventricular assessment, the present review provides an integrated evaluation of both left and right ventricular systolic function beyond ejection fraction, with particular emphasis on their implementation in routine clinical practice.

The literature search for this narrative review was conducted using the PubMed and ScienceDirect databases. The key search terms included left ventricular systolic function, right ventricular systolic function, echocardiography, ejection fraction, ventriculo–arterial coupling, 3D echocardiography, speckle-tracking echocardiography, myocardial work, and cardiac power output. Priority was given to international society guidelines (e.g., ASE/EACVI), meta-analyses, review articles, and pivotal clinical validation or prognostic cohort studies. Additional relevant references were identified through citation tracking and manual screening of reference lists. No strict time restrictions were applied, as landmark earlier studies were also considered essential for physiological and methodological context.

## 2. Physiological Basis of Ventricular Systolic Function

### 2.1. Myocardial Fiber Orientation and Contractile Function of the Left and Right Ventricles

In the left ventricle, three distinct myocardial layers of aggregated cardiomyocytes can be identified. The superficial strands or subepicardial layer, the middle strands or mid-myocardial layer, and the deep strands or subendocardial layer [7]. In this context, the term “oblique fibers” refers to a helical orientation along the longitudinal axis.

The subepicardial layer extends from one ventricle to the other, with fibers arising from the valvular insertions at the base. At the apex of the left ventricle, these fibers spiral inward to form the subendocardial layer, with a similar continuity also observed at the ventricular base [17]. The subepicardial layer accounts for about 25% of the wall thickness. It is composed of left-handed oblique fibers that produce counterclockwise rotation of the apex as viewed from the apex and clockwise rotation of the base [18]. In contrast, the subendocardial layer, comprises less than 20% of the wall thickness. It contains right-handed oblique fibers, which generate clockwise rotation of the apex as viewed from the apex and counterclockwise rotation of the base. The larger radius of the subepicardial region produces higher torque and therefore dominates the rotational mechanics. That results in a global counterclockwise LV rotation near the apex and clockwise rotation near the base during ejection. This apical counterclockwise rotation relative to the basal clockwise rotation is defined as left ventricular twist, expressed in degrees. Torsion refers to the same phenomenon normalized to ventricular length, expressed in degrees per centimeter [19]. It is important to note that during isovolumic contraction (IVC), electrical activation initiated by the endocardial Purkinje fibers leads to subendocardial fiber shortening, which produces an initial untwisting. Subsequently, activation reaches the epicardium, where contraction of the subepicardial fibers, arranged in an opposite left-handed helix, generates systolic twisting during ejection. In diastole, the subepicardial fibers lengthen from base to apex, while the subendocardial fibers lengthen from apex to base, facilitating ventricular recoil and filling. In addition to their role in twist mechanics, subendocardial fibers are the main contributors to longitudinal shortening, whereas subepicardial fibers predominantly support circumferential shortening [19].

The mid-myocardial layer consists of circumferentially oriented fibers, nearly parallel to the plane of the mitral orifice and accounts for approximately 53–59% of the total left ventricular wall thickness [17]. These fibers do not insert into the mitral or aortic valves and do not reach the ventricular apex. The layer is thickest near the base, and gradually thins toward the apex [7]. Importantly, the interventricular septum derives its major structural contribution from this mid-myocardial layer [7]. Contraction of these fibers decreases the minor axis of the ventricle, producing radial shortening [20].

Functionally, the helical orientation of myocardial fibers enables efficient LV contraction. Despite individual myofibers shortening by only 15–20%, their helical arrangement amplifies the effect, producing 35–40% wall thickening and approximately 55% reduction in LV cavity volume [21]. The twisting of subendocardial fibers stores potential energy, which is subsequently released during diastolic recoil [19]. This diastolic untwisting creates a suction effect that lowers LV pressure and enhances early diastolic filling, a mechanism particularly important during exercise when diastolic time shortens [22]. When torsion is impaired, endocardial stress and myocardial oxygen demand increase, reducing the efficiency of systolic function [21]. Abnormal twist mechanics are observed in several pathological conditions, including hypertrophic and dilated cardiomyopathy, ischemic heart disease, and HF, where disruption of the normal transmural fiber balance reduces contractile efficiency and compromises diastolic function [23,24]. While these characteristics define LV structure and function, the right ventricle exhibits distinct anatomical and mechanical features.

In the right ventricle, a distinct mid-myocardial layer cannot be identified, as only subepicardial and subendocardial layers are present. The subepicardial layer, accounting for about 25% of the wall thickness, is composed mainly of circumferential fibers that run parallel to the atrioventricular groove and extend across both ventricles. Within the right ventricle, these fibers are more circumferentially oriented than in the left ventricle. The subendocardial RV layer consists of longitudinal fibers that pass through the apex toward the papillary muscles, tricuspid annulus, and outflow tract, remaining continuous with the septum [4]. Functionally, this fiber arrangement explains the characteristic contraction mechanics of the RV. The predominance of longitudinally oriented subendocardial fibers accounts for nearly 75% of RV systolic shortening, while the more circumferential orientation of subepicardial fibers contributes to the bellows-like inward motion of the free wall [25]. RV contraction occurs sequentially, beginning in the inlet and trabeculated regions and ending in the outflow tract or infundibulum about 25–50 ms later, with infundibular contraction lasting longer than that of the inlet. Three main mechanisms contribute to RV systolic function. Inward motion of the free wall, known as the bellows effect, longitudinal shortening that draws the tricuspid annulus toward the apex, and traction on the free wall due to LV contraction. Shortening is greater longitudinally than radially, and twisting or rotational movements contribute minimally to RV ejection. Due to its slightly larger end-diastolic volume (EDV) compared with the left ventricle and its greater surface-to-volume ratio, the right ventricle, requires less inward motion to eject the same stroke volume (SV) [26]. The apex itself contributes little to ejection, as blood flow is directed along the septum in a streamlined fashion from inflow to outflow [27]. Importantly, septal contraction, supported by the mid-myocardial architecture of the left ventricle, provides a major contribution to RV SV, highlighting the close systolic interdependence of the two ventricles [10,26].

### 2.2. Ventricular Load Conditions: Preload and Afterload

Preload describes the degree of myocardial stretch or distention before contraction has started [28]. Indices commonly used to estimate preload include end-diastolic pressure (EDP) and EDV. End-diastolic wall stress, derived from the Law of Laplace, is considered the most physiologically relevant measure. However, it is less practical for routine clinical assessment. End-diastolic sarcomere length, although conceptually important, remains an experimental index [29,30]. As preload increases, sarcomere length also increases, improving the geometric overlap between actin and myosin filaments and allowing more cross-bridge formation, which enhances active tension generation [31]. This behavior underlies the ascending limb of the length–tension relationship, where sarcomere lengths rising from approximately 1.8 μm to 2.2 μm optimize filament interaction and force production [32,33,34]. Moreover, greater sarcomere length enhances myofilament Ca^2+^ sensitivity, so that for a given intracellular Ca^2+^ concentration, more cross-bridges form and greater force is generated [35,36]. However, this relationship is not limitless. Beyond an optimal length, further stretch reduces filament overlap and does not increase force, yielding a plateau. Consequently, SV rises with increasing preload until this limit is reached, which represents the physiological basis of the Frank–Starling law also known as heterometric autoregulation [29].

Afterload refers, in a broad sense, to the forces opposing LV ejection [28]. Common indices of afterload include aortic pressure, total peripheral resistance, and myocardial wall stress during systole. Myocardial wall stress during systole is considered the most accurate representation of afterload. The instantaneous wall stress during systole, as described by the Law of Laplace, quantifies afterload at a given moment. The integral of these instantaneous values over time, known as integrated wall stress, represents the total load imposed on the myocardium throughout the entire systolic phase, including the IVC period. In contrast to preload, which is defined as a single instantaneous value at end-diastole, afterload reflects a dynamic load [30,37]. As systolic wall stress increases, fractional shortening and fiber shortening velocity decrease, reflecting the increased workload on the ventricle [38,39]. Conversely, if afterload were reduced to zero, the myocardium would contract at its maximum velocity (Vmax), representing its intrinsic contractile capacity independent of loading conditions [40]. Additionally, a sustained increase in afterload activates the Anrep effect, also known as homeometric autoregulation, which enhances myocardial contractility. This response involves stimulation of the Na^+^/H^+^ exchanger and an increase in intracellular Na^+^. The latter promotes Ca^2+^ influx via the Na^+^/Ca^2+^ exchanger, resulting in greater sarcoplasmic reticulum Ca^2+^ loading and increased Ca^2+^ availability for contraction [41]. Understanding afterload is essential, as it not only influences ventricular performance but also modulates the manifestation of systolic function in both physiological and pathological states [42].

### 2.3. End-Systolic Elastance, Arterial Elastance, and Ventriculo–Arterial Coupling

Elastance is defined as the change in pressure for a given change in volume, and the higher the elastance, the stiffer the ventricular wall [32]. End-systolic elastance (Ees) reflects the maximum pressure that can be generated by the ventricle at any given end-systolic volume (ESV). It is considered a load-independent index of myocardial contractility, representing the intrinsic strength of the ventricular myocardium. This intrinsic contractility depends on factors such as the amount of calcium released to the myofilaments, the affinity of myofilaments for calcium, and the availability of myofilaments participating in the contraction process. Compliance, the inverse of elastance, is defined as the change in volume for a given change in pressure. Arterial elastance (Ea) is an index of afterload that reflects the overall properties of the arterial system and is closely related to total peripheral resistance [43]. Finally, the concept of ventriculo–arterial (VA) coupling describes the ability of the left or right ventricle to respond appropriately to the arterial load [44]. The term coupling itself is borrowed from engineering, where it refers to a device used to connect two shafts together at their ends for the purpose of transmitting power, ensuring uninterrupted continuity and coordinated function without disconnection during operation [45]. Similarly, in cardiovascular physiology, VA coupling reflects the interaction between the intrinsic contractile properties of the ventricle and the afterload imposed by the arterial system [46].

### 2.4. Left and Right Ventricular Adaptation to Load

The left ventricle tolerates acute increases in afterload remarkably well. Even large, abrupt rises of 40–50 mmHg in aortic pressure have little immediate effect on cardiac output. When afterload increases acutely, ejection is limited and EDV rises, enhancing force generation and pressure in the subsequent beat through the Frank–Starling mechanism. Over seconds to minutes, a slower adaptation occurs, with increased inotropic state and larger Ca^2+^ transients, a phenomenon known as the Anrep effect [28]. In the setting of chronic afterload elevation, such as sustained arterial hypertension, long-term adaptations occur, including changes in gene expression, extracellular matrix remodeling, and myocyte hypertrophy. These processes lead to increased wall thickness as a compensatory response to pressure overload. However, the pattern of hypertrophy that develops is not uniform and can vary depending on multiple factors [13]. Increased preload, corresponding to volume overload, enhances SV through the Frank–Starling mechanism, but sustained elevation can eventually lead to chamber dilation and increased wall stress. It is worth mentioning that persistent hemodynamic load due to sustained pressure or volume overload can eventually lead to VA uncoupling [47].

The right ventricle is highly sensitive to changes in afterload, and its adaptive capacity differs markedly from that of the left ventricle. Even modest acute increases in afterload, often in the range of 15–20 mmHg above baseline, can markedly impair RV performance. Its thin wall and low-pressure system fail to generate sufficient pressure, causing rapid RV failure with reduced SV and cardiac output [48]. Chronic increase in RV afterload, corresponding to pressure overload, is initially met by the Anrep effect, which enhances contractility to preserve SV. Wall thickness increases to normalize wall stress. As overload persists, the Frank-Starling mechanism is activated, with mild RV dilation and increased EDV augmenting SV. Heart rate also rises to help maintain cardiac output. These mechanisms are initially adaptive and support RV–pulmonary arterial (PA) coupling. Over time, they may become maladaptive, leading to increased wall stress, higher oxygen demand, leftward septal shift, reduced cardiac output, and RV-PA uncoupling [48,49]. Increased preload, corresponding to volume overload, is generally better tolerated by the right ventricle. This tolerance is largely attributed to the high compliance and bellows-like geometry of the right ventricle [26]. During acute increases in preload, for example, with rapid volume infusion, postural changes, or exercise, the Frank–Starling mechanism enhances SV without a significant rise in pulmonary arterial pressure. The ability to augment SV during acute preload elevation relies on a compliant pulmonary circulation. In pulmonary arterial hypertension, this preload reserve is diminished due to a disproportionate rise in systolic and mean pulmonary arterial pressures [50]. Chronic elevation of preload, as in tricuspid regurgitation (TR) or atrial septal defect, leads to progressive RV dilation, increased wall stress, and ultimately a transition to a maladaptive state [48].

## 3. Conventional Echocardiographic Indices

Echocardiography plays a pivotal role in the measurement of systolic function in everyday clinical practice. Table 1 provides a summary of the echocardiographic parameters, their challenges, and their prognostic value.

### 3.1. Left Ventricular Systolic Function

The conventional assessment of LV systolic function has traditionally relied on metrics such as the LVEF, most commonly measured by Simpson’s biplane method or, more accurately, by 3D echocardiography. Other echocardiographic indices, including fractional shortening (FS), Doppler-derived dP/dt, and tissue Doppler imaging (TDI)-derived systolic velocity (S′), have also been employed.

LVEF is defined as the ratio of LV SV to LV EDV [51]. Consequently, it is influenced by physiological factors that affect LV SV, including preload, afterload, contractility, lusitropy, heart rate, and the synchrony of LV ejection [52]. At the same time, it is also determined by structural variables that influence LV EDV, such as LV size, shape, and wall thickness [52]. The decline in LVEF may result either from an increase in LV EDV or from a reduction in LV SV [53]. This raises the question of whether, in the longitudinal follow-up of patients with HF, monitoring LV EDV and SV might provide more meaningful information than relying solely on LVEF [1,54,55,56]. Available data indicate that SV carries important prognostic implications not only in HF but also in valvular heart disease [57]. LVEF may vary independently of true contractile function, as patients with hypertrophic cardiomyopathy and dilated cardiomyopathy can have markedly different LV EDVs, resulting in different EFs despite similar SV or intrinsic contractility [53]. Evidence from both clinical studies and mathematical modeling indicates that in LV hypertrophy, LVEF can remain preserved or even appear normalized, despite reduced longitudinal fiber shortening and a concomitant fall in SV. This paradox further illustrates the limitations of LVEF as a measure of LV systolic function [58,59].

**Figure 1:**
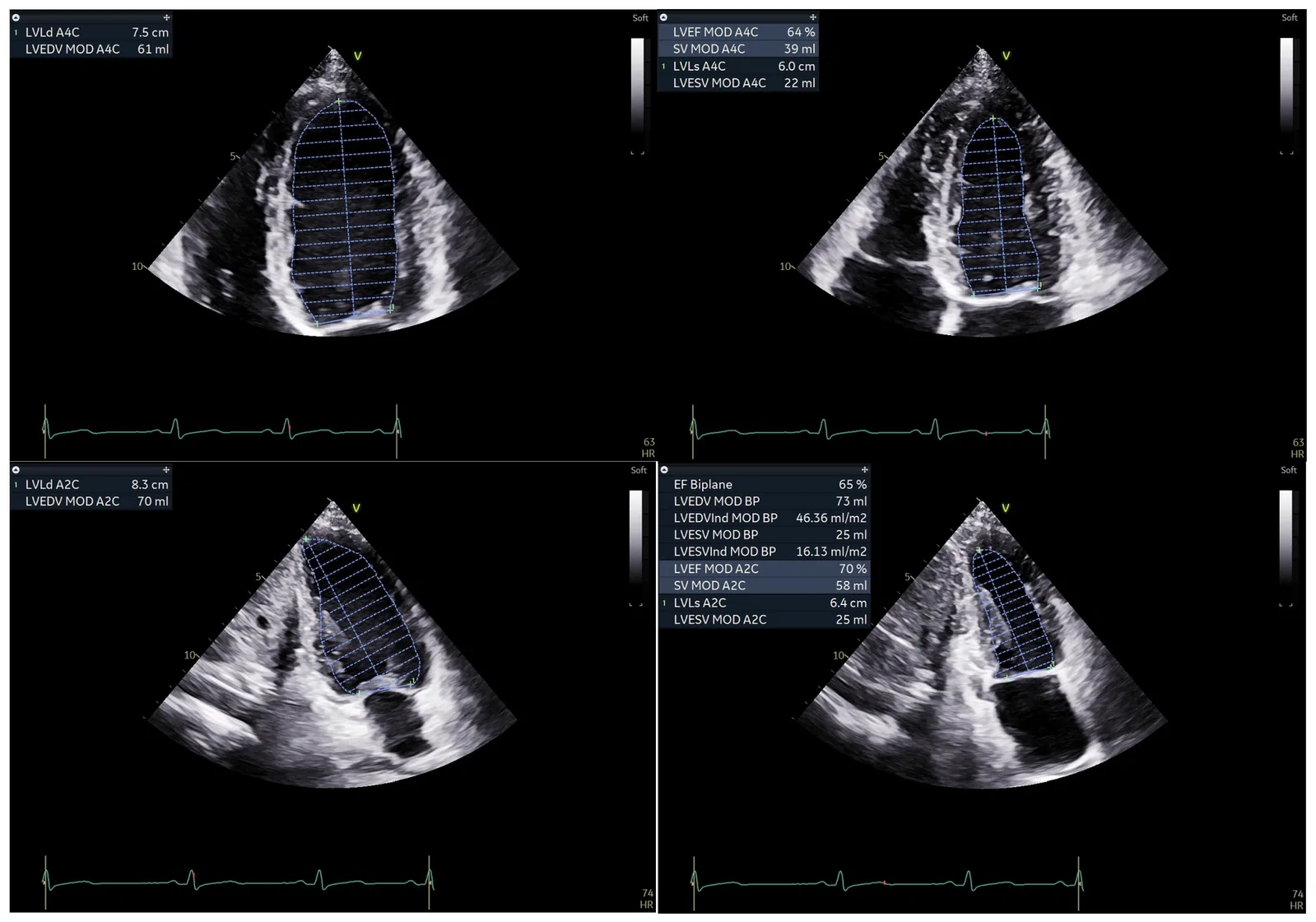
Calculation of LVEF using Simpson’s method of disks in 4- and 2-chamber views, showing end-diastolic and end-systolic frames with endocardial delineation. Analysis was performed during sinus rhythm.

The most reliable 2D method for calculating LVEF in routine clinical practice is Simpson’s biplane method of disks (Figure 1). This technique is based on the summation-of-disks principle where the LV cavity is divided into a series of elliptical cylinders reconstructed from the apical four- and two-chamber views. For the method to be valid, several assumptions must hold true [20,60]. The apical views should be orthogonal and correctly aligned with the true long axis of the ventricle, the basal plane should be perpendicular to this axis, and each cross-sectional slice of the LV is assumed to have an elliptical shape with its major and minor axes contained within the imaging planes [61]. The most common limitation is foreshortening where failure to include the true apex results in underestimation of volumes [62]. Additional sources of error include basal slanting, misalignment of the imaging planes relative to the true axes of the ellipses, and geometric heterogeneity in remodeled ventricles [61]. Furthermore, the accuracy of Simpson’s method is highly dependent on image quality and precise endocardial border delineation, making it less robust in patients with poor acoustic windows or irregular LV geometry [63]. In this context, the administration of contrast agents has proven effective in improving LVEF determination in patients with challenging acoustic windows [64].

Assessment of LVEF is strongly influenced by the imaging modality, and even when the same technique is applied, there is marked intraobserver and interobserver variability. In practice, repeated measurements of LVEF in the same patient by experienced echocardiographers may differ by around 7%, and the variability is even higher in everyday clinical settings. In the context of clinical trials, when echocardiograms are re-analyzed by core laboratories using standardized protocols, discrepancies of up to 15% compared with local investigators are frequently observed, even when reviewing identical images. The accuracy of LVEF is further limited by its dependence on operator skill, image quality, and loading conditions [2]. Consequently, a considerable number of patients with LVEF in the range of 40–50% may be reclassified as having values either below 40% or above 50% if the measurement is repeated [2,65]. Many of the limitations of 2D-derived LVEF can be overcome by acquiring a 3D dataset of the left ventricle [66]. Additionally, studies have consistently demonstrated that echocardiography underestimates LVEF when compared with the advanced imaging capabilities of CMR [67]. As a result, CMR is regarded as the reference standard for non-invasive quantification of LV volumes and LVEF, providing superior accuracy and reproducibility [3].

FS is also a widely used echocardiographic measure of LV systolic function. FS can be derived from 2D-guided M-mode imaging or, preferably, from linear measurements obtained directly from 2D images [11]. Endocardial fractional shortening (eFS) is conventionally calculated as the percentage reduction in LV internal diameter between end-diastole and end-systole. This index reflects inward motion of the endocardium but does not necessarily represent true myocardial fiber shortening. In LV hypertrophy (LVH), the thickened wall exaggerates endocardial displacement, often yielding normal or even supranormal eFS despite impaired contractility [59]. To overcome this limitation, midwall fractional shortening (FSmw) was introduced [68]. FSmw estimates midwall fiber shortening by modeling the left ventricle as two concentric cylindrical shells of equal thickness at end-diastole, assuming constant wall volume throughout the cardiac cycle [69,70]. Both eFS and FSmw, however, are derived from linear M-mode measurements and share intrinsic limitations [11]. They are load-dependent, assume uniform geometry and homogeneous motion, and are prone to errors from foreshortening, poor acoustic windows, and regional wall motion heterogeneity [71]. An increasingly adopted approach is the stress-corrected FSmw, which provides a more refined assessment of systolic performance, particularly valuable in LVH [13]. Unlike conventional eFS, this parameter offers a more accurate representation of myocardial contractility and is relatively independent of afterload, as it incorporates LV peak systolic pressure estimated from brachial-cuff systolic blood pressure. Nevertheless, both FSmw and stress-corrected FSmw remain infrequently used in routine clinical practice, likely due to the absence of automated calculation algorithms in standard echocardiographic systems. These indices are mainly applied in research settings, where dedicated quantitative and statistical models are used to derive and validate midwall mechanics, often necessitating specialized software [68].

LV contractile performance can be assessed noninvasively by calculating the rate of pressure rise during IVC, expressed as dP/dt. Two echocardiographic methods are commonly used. The first relies on the continuous-wave Doppler profile of a mitral regurgitation (MR) jet, in which the time required for the velocity to increase from 1 to 3 m/s is measured, which corresponds to a 32 mmHg pressure rise according to the modified Bernoulli equation. dP/dt is then obtained as 32 divided by this time interval (mmHg/s) [72]. The second approach estimates mean dP/dt during IVC by dividing the pressure difference between brachial diastolic pressure and estimated LV end-diastolic pressure by the IVC time (IVCT) derived from M-mode echocardiography and phonocardiography [73]. Values below 1000 mmHg/s are considered abnormal, while severely impaired systolic function is indicated when dP/dt falls below 500 mmHg/s [74]. The Doppler-derived dP/dt from the MR jet provides a useful index of LV systolic performance and shows good correlation with invasive measures of contractility, although its accuracy is strongly influenced by loading conditions and the severity of mitral regurgitation [75,76,77].

TDI-derived S′ is a measurement used to assess the longitudinal systolic function of the left ventricle [78]. The measurement is typically obtained by placing the pulsed-wave Doppler sample volume in the myocardial region adjacent to the mitral annulus, ensuring that it remains within the ventricular myocardium rather than the atrial wall at end-systole. Alternatively, color TDI can be used, allowing velocity measurements for each myocardial pixel along the ultrasound beam. Although TDI data can be acquired from all apical views, it is most commonly obtained in the apical four-chamber view, focusing on the basal lateral and septal segments. Because this method is angle-dependent, the annular motion should be aligned parallel to the M-mode line, which may require slightly nonstandard apical views. A good correlation has been demonstrated between LVEF and average S′ in patients with both normal EF and systolic dysfunction [79,80]. An average S′ value below 8 cm/s correlates well with LVEF between 30% and 50%, whereas a value below 6 cm/s is indicative of LVEF less than 30% [80]. However, reduced S′ is not a specific marker of impaired longitudinal systolic function in patients with severe mitral annular calcification or following mitral valve repair or replacement, as the mechanical restriction of annular motion limits basal myocardial excursion [20]. Regional variations in longitudinal performance can be evaluated by positioning the sample volume at different myocardial levels, from base to apex, or by using color TDI, which also allows off-line analysis [81]. As with LVEF, S′ velocity is influenced by loading conditions, being both preload- and afterload-dependent, and should therefore be interpreted in the context of the patient’s haemodynamic status [82]. Nevertheless, the S′ velocity is valuable for detecting subclinical myocardial dysfunction even before changes in EF occur, in conditions such as arterial hypertension, coronary artery disease, diabetes mellitus, and hypertrophic cardiomyopathy [83]. Furthermore, it provides prognostic information in patients with coronary artery disease and HF [84,85].

### 3.2. Right Ventricular Systolic Function

Echocardiographic evaluation of right ventricular (RV) systolic function requires an integrated approach, as no single parameter fully captures the complexity of RV mechanics. Commonly used indices include tricuspid annular plane systolic excursion (TAPSE), TDI-derived S′ of the tricuspid annulus, the Doppler-derived dP/dt from the TR jet, fractional area change (FAC), the RV index of myocardial performance (RIMP) assessed with pulsed-wave Doppler or TDI, and right ventricular ejection fraction (RVEF), preferably derived from 3D echocardiography [86,87].

TAPSE is a simple, reproducible, and widely used linear echocardiographic index of RV systolic function obtained with M-mode imaging [88]. It is measured in an RV-focused apical four-chamber view by aligning the M-mode cursor through the lateral tricuspid annulus, and it represents the longitudinal displacement of the annulus from end-diastole to end-systole, expressed in millimeters [20]. TAPSE strongly correlates with RVEF measured by radionuclide angiography and also correlates well with RV FAC. It also has low interobserver variability, which has established its role as a practical surrogate marker of RV function [89,90,91]. An abnormality threshold of <17 mm is generally accepted. The major strength of TAPSE lies in its simplicity, feasibility, and validation in large patient cohorts [92,93,94,95,96]. The main limitation of TAPSE is the assumption that all motion of the anterolateral tricuspid annulus originates from RV longitudinal shortening. It is angle-dependent and may be affected by translational motion of the heart within the chest, respiratory variation, or pericardial effusion. Moreover, TAPSE is load-dependent, and in the presence of severe TR values may be falsely elevated, while in early stages of RV dysfunction TAPSE can still appear within the normal range. Despite these caveats, TAPSE remains a robust and guideline-endorsed parameter for routine clinical evaluation of RV systolic function [20].

Another widely used Doppler-based surrogate of RV longitudinal systolic function is the S’ of tricuspid annulus. S′ represents the peak systolic velocity of the lateral tricuspid annulus, reflecting the speed (distance per unit time) at which the annulus moves toward the RV apex during systole [97]. It is measured in an RV-focused apical four-chamber view, with the sample volume placed at the lateral tricuspid annulus and acquired at high frame rates [97]. Validation studies have demonstrated good correlation of tricuspid S′ with RVEF measured by radionuclide angiography and CMR [98]. In fact, tricuspid S′ correlated better with CMR-derived RVEF than FAC and TAPSE when RVEF was normal to mildly abnormal (<50%), but it was second to FAC when CMR-derived RVEF was severely reduced (<30%) [99]. In clinical practice, thresholds of <9.5 cm/s for pulsed-wave Doppler tricuspid S′ and <6 cm/s for color Doppler tricuspid S′ are considered abnormal. Similar to TAPSE, this index is angle-dependent and may be influenced by overall cardiac motion or suboptimal visualization of the lateral tricuspid annulus. Despite these limitations, tricuspid S′ is a simple, reproducible, and guideline-endorsed parameter for the routine assessment of RV systolic performance [20,87].

An additional Doppler-derived parameter of RV systolic function is the RV dP/dt. This index quantifies the RV systolic change in pressure over time using the TR jet on continuous-wave Doppler. In practice, dP/dt of the RV is calculated by measuring the time it takes for the TR jet velocity to rise from 1 to 2 m/s [100]. According to the simplified Bernoulli equation, this corresponds to a pressure increase from 4 to 16 mmHg, giving a difference of 12 mmHg. dP/dt is then calculated as 12 divided by this time interval, expressed in mmHg/s. A value < 400 mmHg/s is generally considered abnormal [20,87]. The main limitations of RV dP/dt are its load dependency and its reliance on a well-defined TR spectral envelope, with reduced accuracy in severe TR due to rapid pressure equalization. Although it correlates well with invasive measures of contractility, the lack of robust normative data prevents its recommendation for routine clinical application [15,20].

The RV index of myocardial performance (RIMP), also known as the Tei index, is an indirect parameter that integrates systolic and diastolic time intervals to assess RV global function [101]. It is calculated as the ratio of non-ejection time to ejection time (ET), where non-ejection time is the sum of IVCT and isovolumic relaxation time (IVRT). With pulsed-wave spectral Doppler, the necessary intervals are obtained from separate recordings of tricuspid inflow and RV outflow, which means that different cardiac cycles are used. In contrast, tissue Doppler imaging allows IVCT, IVRT, and ET to be measured from the same tracing of the lateral tricuspid annulus within a single cardiac cycle, improving feasibility and reproducibility [15]. Cut-off values differ depending on the technique, with an upper reference limit of 0.40 for the spectral Doppler method and 0.55 for the tissue Doppler method [87]. Although RIMP has shown prognostic value in pulmonary hypertension, congenital heart disease, RV infarction and hypertrophic cardiomyopathy, its limitations include dependence on good image quality, difficulty in clearly distinguishing IVCT and IVRT, and reduced reliability in patients with irregular rhythm or elevated right atrial pressure [87,102,103,104,105].

Another classical echocardiographic index for the evaluation of RV systolic function is FAC. It is measured from an RV-focused apical four-chamber view by tracing the endocardial border at end-diastole and end-systole, carefully excluding the heavy trabeculations of the right ventricle. FAC is calculated as the difference between end-diastolic and end-systolic areas divided by the end-diastolic area, expressed as a percentage. Unlike volumetric techniques applied to the left ventricle, FAC is a purely 2D fractional calculation and does not rely on geometric assumptions of 3D shape [20]. An FAC value < 35% is commonly used as a threshold for abnormality according to current guidelines [11,14,15]. The parameter has been validated against CMR-derived RVEF, demonstrating good correlation, and has been associated with adverse outcomes, including HF, sudden death, stroke, and all-cause mortality in patients after myocardial infarction as well as cardiac death and all-cause mortality in patients with chronic HF [106,107,108,109,110,111,112]. The main advantages of FAC are its simplicity and feasibility, similar to TAPSE, which explains its recommendation as a routine measure of RV function. However, as a 2D measurement, it does not fully capture global RV performance or provide a true estimate of RVEF. Moreover, FAC provides only limited assessment of the RV outflow tract (RVOT) contribution to RV contraction and, like other conventional indices, may be influenced by loading conditions, in particular, significant TR may lead to misleadingly preserved or increased values. Its accuracy depends heavily on image quality and adequate visualization of the RV free wall, particularly at the apex, where the confluence of trabeculations with the moderator band and near-field artifacts can obscure the true endocardial border [20,87].

Efforts have also been made to quantify RV systolic function by calculating RVEF. Τhe accuracy of this approach, however, is strongly dependent on the method used [11,14]. Early attempts relied on 2D echocardiographic methods. The area-length technique relies on simplified assumptions of RV geometry, such as ellipsoidal or pyramidal models, which consistently underestimate true volumes. The disk summation method, adapted from the biplane Simpson’s technique, also underestimates RV volume because it primarily includes the ventricular body while excluding the inflow and outflow tracts [87]. Compared with CMR, which remains the reference standard for volumetric assessment, both of these 2D methods demonstrate limited accuracy and lower reference values of RVEF [11]. The use of 2D echocardiography for the calculation of RVEF is not supported by current guidelines, in contrast to LVEF, where the biplane Simpson’s method remains standard practice [11,14]. When assessed with 3D echocardiography, however, RVEF becomes a more reliable and clinically meaningful parameter, but this will be addressed in a subsequent section [113,114].

## 4. Novel Echocardiographic Indices

For practical guidance, key measurement considerations for 3D echocardiography and speckle tracking echocardiography (STE) are summarized in Table 2.

### 4.1. Three-Dimensional Echocardiography

Many of the limitations of 2D-derived LVEF can be addressed by acquiring a 3D dataset of the left ventricle. This enables tracing of the compacted myocardium along the entire endocardial surface and minimizes geometric assumptions [66]. The 3D approach also reduces ventricular foreshortening and is therefore considered technically superior to 2D methods [115]. Studies have demonstrated that 3D-derived LVEF provides incremental prognostic value compared with 2D measurements, particularly in patients at high cardiovascular risk [116,117]. To benefit from 3D quantification, image quality must be optimized, as spatial and temporal resolution remain lower than in 2D imaging. During 3D volume acquisition, all myocardial segments are obtained from the same scanning point, which prevents the operator from eliminating artifacts arising from the ribs or lungs through minor transducer movements, as can be done in 2D imaging. Therefore, 3D datasets should be acquired under visual control to ensure that all apical and parasternal views typically used in 2D echocardiography are adequately represented in the recorded volume. Likewise, the tracking line generated for LV quantification must be carefully reviewed in the reconstructed views to confirm accurate endocardial border delineation [20]. Available 3D software includes automated border detection algorithms that facilitate volume measurements. This feature substantially reduces interobserver variability by minimizing the need for manual contouring, although the accuracy of the automatically generated endocardial borders must always be visually verified and corrected when necessary [115]. Studies have consistently shown that 3D echocardiography yields higher and more accurate SV values compared with 2D methods. This difference mainly reflects the reduced foreshortening of the ventricle. Moreover, 3D imaging, especially when using a 3D-derived left ventricular outflow tract (LVOT) area, can depict the true geometry of both the left ventricle and the outflow tract. As a result, SV measurements obtained by 3D echocardiography closely match those derived from CMR [118,119,120]. However, LVEF typically remains similar between the two methods, since proportional differences in both volumes cancel out in percentage calculation [121,122]. Normal reference ranges for 3D-derived LVEF are not yet fully established [20]. Of note, in the presence of significant MR, LVEF may be misleading for the assessment of LV systolic function. In this setting, the total SV (EDV–ESV) includes both the forward LV SV and the regurgitant volume, which may lead to overestimation of LV pump performance regardless of whether LVEF is measured by 2D or 3D echocardiography. Therefore, forward LVEF (forward LV SV/LVEDV) may provide a more reliable estimate of LV function than global LVEF in patients with MR [123].

**Figure 2:**
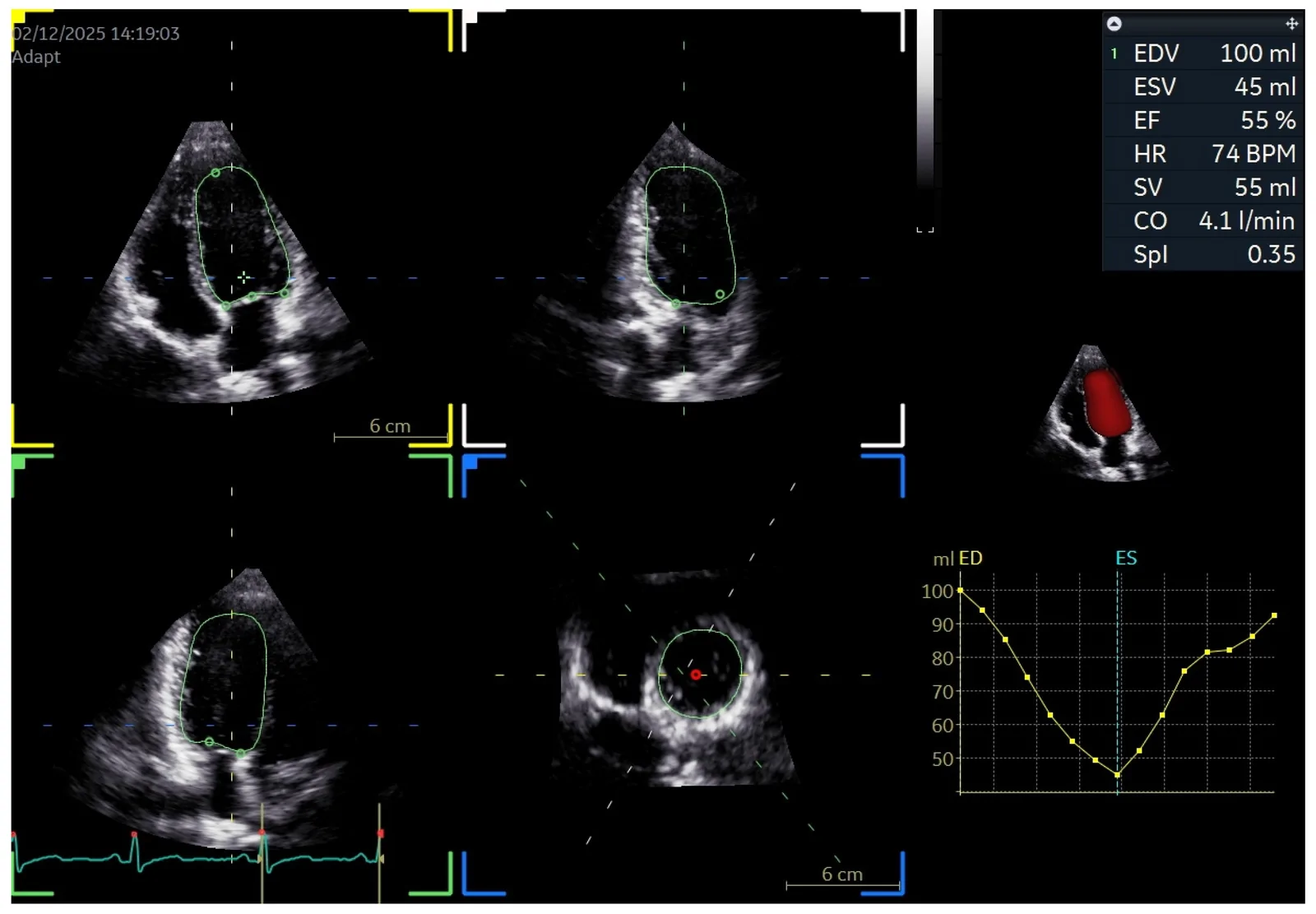
3D echocardiographic reconstruction of the left ventricle illustrating automated volumetric analysis and calculation of end diastolic volume (EDV), end systolic volume (ESV), left ventricular ejection fraction (EF), stroke volume (SV) and cardiac output (CO) together with the corresponding left ventricular volume–time curve. Analysis was performed during sinus rhythm using a full-volume 3D dataset acquired at an adequate volume rate.

In conclusion, although 3D echocardiography does not markedly alter LVEF as a percentage, it provides more accurate, objective, and reproducible measurements of LV volumes and geometry. It is therefore technically and prognostically superior, provided that image quality and operator expertise are adequate, although it remains a load-dependent technique (Figure 2).

**Figure 3:**
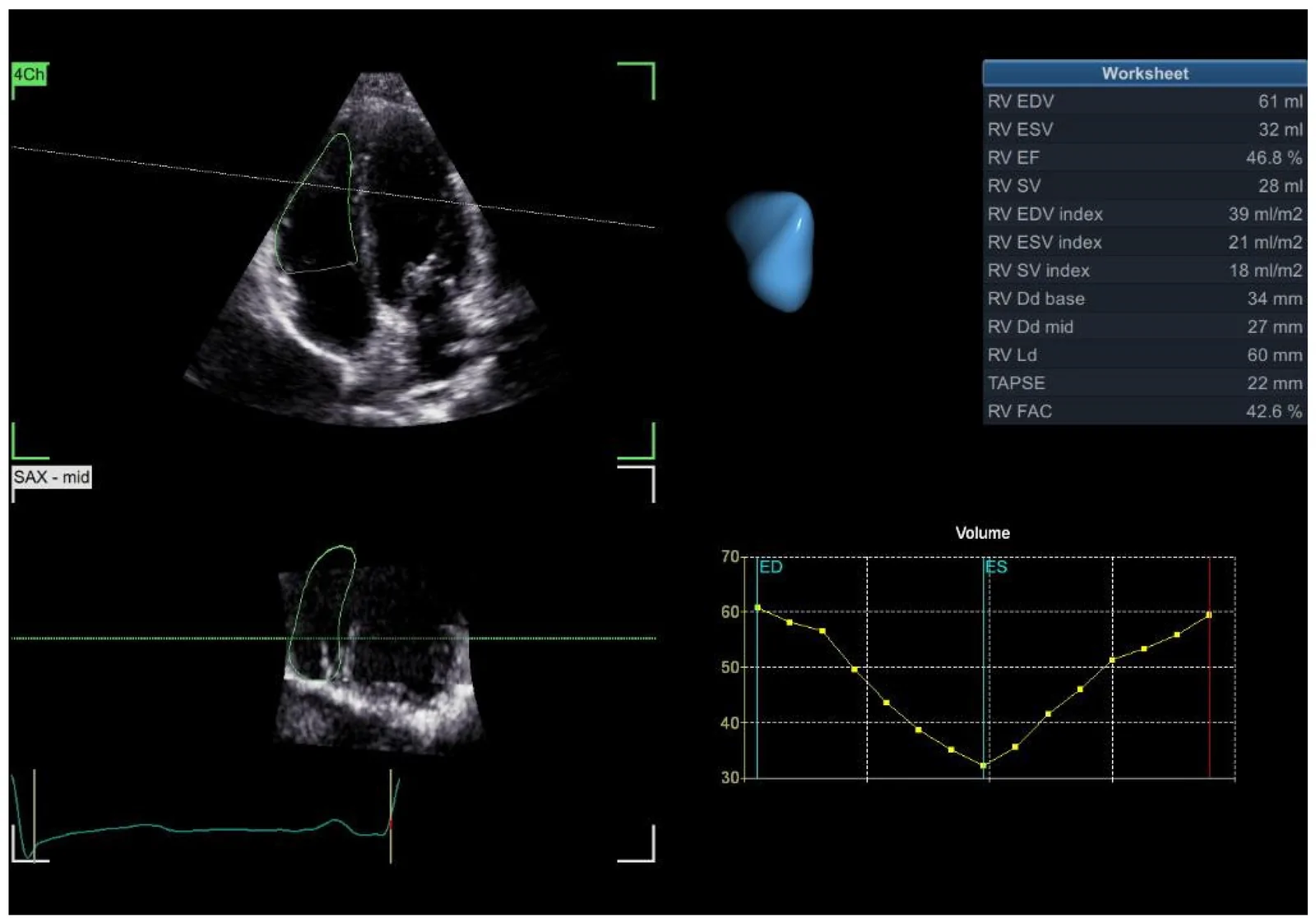
3D echocardiographic assessment of the right ventricle. Automated volumetric analysis demonstrates calculation of right ventricular end-diastolic volume (RV EDV), end-systolic volume (RV ESV), stroke volume (RV SV), and right ventricular ejection fraction (RVEF) together with the corresponding right ventricular volume–time curve. Analysis was performed during sinus rhythm using a full-volume 3D dataset acquired at an adequate volume rate.

3D echocardiography-derived RVEF is considered the preferred echocardiographic method for quantitative assessment of RV systolic function, because it overcomes the geometric assumptions inherent in 2D imaging [14]. In contrast to 2D echocardiography, which systematically underestimates RV volumes and relies on geometric assumptions, it cannot reliably quantify RVEF. By overcoming these limitations, 3D echocardiography enables direct volumetric reconstruction of the complex RV shape. Real-time 3D RV imaging shows good agreement with CMR and demonstrates acceptable reproducibility, providing the first robust echocardiographic method for RV volume and RVEF assessment. However, in the presence of significant TR, RVEF may be misleading, as the total RV SV includes both forward and regurgitant flow [124]. In addition, most validation data derive from selected cohorts with optimal image quality, and additional studies are required in patients with markedly dilated or remodeled right ventricles [114] (Figure 3).

Practical acquisition considerations for 3D echocardiography are summarized in Table 2.

### 4.2. Ventriculo–Arterial Coupling

A proper assessment of the interaction between the ventricle and the arterial system requires that both ventricular systolic performance and systemic arterial properties be expressed in comparable units. Sunagawa and colleagues established this framework by defining ventricular contractility through the end-systolic pressure–volume relationship (ESPVR), which represents the linear relation connecting all end-systolic pressure–volume points obtained under varying loading conditions. The slope of this relationship, termed end-systolic elastance (Ees), quantifies the contractile stiffness of the ventricle. In contrast, arterial load is represented by the effective arterial elastance (Ea) [125]. Both parameters share the same dimensional units of elastance, expressed as the change in pressure over the change in volume (ΔP/ΔV), with units of mmHg/mL. While Ees reflects the intrinsic contractile strength of the ventricle, Ea represents the combined extracardiac forces opposing ventricular ejection, summarizing the total arterial load. The ratio Ea/Ees therefore provides a simplified yet powerful index for quantifying ventriculo–arterial coupling [126].

The ventricular pressure–volume (PV) loop offers a comprehensive description of cardiac pump behavior. If simultaneous pressure and volume measurements were available for both ventricles, separate PV loops could illustrate their distinct mechanical features. The RV loop typically shows much lower pressure generation compared with the LV and a more triangular shape, as ejection occurs mainly after peak pressure due to the low pulmonary afterload. When preload is gradually reduced, for instance, by transient occlusion of venous return, multiple PV loops can be obtained at different filling levels. This experimental approach, often performed in animal models by transient inferior vena cava occlusion, allows the ventricle to contract against progressively lower preload, thereby defining a set of end-systolic pressure–volume points [127,128,129,130]. Alternatively, the ESPVR can also be derived by transiently increasing afterload, for example through aortic constriction or pharmacologic vasoconstriction, to generate end-systolic points under higher loading conditions [131]. The straight line connecting these points defines the ESPVR, which describes the linear relationship between end-systolic pressure (Pes or ESP) and ESV or Ves across varying loading conditions. Its intercept with the volume axis (V_0_) corresponds to the theoretical point where contraction would occur at zero preload, representing the unstressed ventricular volume. The slope of ESPVR, termed Ees, quantifies the maximal contractile stiffness of the ventricle and remains largely independent of loading conditions [132]. Ees is therefore regarded as the most representative and load-independent index of ventricular contractility [40,125]. Mathematically, Ees is defined as the ratio of Pes to the difference between Ves and the theoretical volume at zero preload or V_0_. The Ees thus reflects the intrinsic contractile state of the myocardium, and variations in contractility lead to proportional changes in Ees without confounding effects of preload or afterload [133].

Conversely, the end-diastolic pressure–volume relationship (EDPVR), fitted through the diastolic points, reflects the passive viscoelastic properties of the ventricle during filling and describes its compliance which is the ability of the myocardium to accommodate changes in volume with minimal pressure rise [134]. Both ESPVR and EDPVR derive from the time-varying elastance model, which conceptualizes the heart as a dynamic elastic chamber whose stiffness changes throughout the cardiac cycle [135].

The concept of Ea derives from the Windkessel model, which describes the arterial system as a combination of resistance (R) and compliance (C). Although the complete model includes time-dependent terms related to systole and diastole, these parameters are difficult to measure directly. By simplifying the basic hemodynamic relationship between pressure, flow, and resistance, expressed as ΔP = Q × R. It is further assumed that ventricular and arterial pressures are approximately equal during ejection, venous pressure is negligible, and mean flow can be approximated by SV × HR. Under these conditions, Ea can be expressed clinically as the ratio of ESP or Pes to SV (P_es_/SV) [136]. This approximation does not equate Ea with afterload per se but represents an integrated, lumped parameter that encapsulates both the resistive and compliant components of the arterial system, providing a practical macroscopic descriptor of the total arterial load opposing ventricular ejection [137,138]. In the PV loop, Ea can be graphically represented as a straight line connecting the Pes with the EDV on the volume axis, a line that extends downward toward the base of the PV loop [139]. Ea therefore does not represent a physical stiffness of any vascular wall, but rather a virtual linear PV relationship that summarizes the total arterial load encountered by the ventricle during ejection [140].

Direct measurement of ventricular PV loops is technically demanding and rarely feasible in clinical settings. Therefore, several simplified methods have been developed to estimate Ees and Ea without invasive procedures. The concepts of Ea and Ees can be applied to both the LV and RV, reflecting the interaction between each ventricle and its respective arterial system. For the LV, Pes is commonly approximated as 90% of the brachial or radial systolic arterial pressure, or alternatively from the aortic dicrotic notch pressure when an invasive arterial waveform is available [141,142,143,144]. However, since mean arterial pressure (MAP) remains relatively constant throughout the arterial tree and is less affected by pulse wave distortion, the Ea estimate based on MAP/SV provides a more stable and reliable surrogate across different hemodynamic conditions [145]. In the RV, Pes is commonly approximated by the mean pulmonary arterial pressure (mPAP) [146]. The single-beat method proposed by Kelly et al. defines Ees as the ratio of Pes to ESV, assuming a negligible V_0_ [147]. However, this approximation should be regarded as a crude surrogate, since assuming a fixed V_0_ renders the Pes–ESV slope load-dependent [148]. In contrast, the single-beat method developed by Chen et al. employs a normalized elastance–time function (END(avg)) derived from population data and incorporates noninvasive pressure and echocardiographic measurements through dedicated mathematical algorithms [149,150]. Although this method allows individualized estimation of Ees without altering loading conditions, it relies on the assumption that the END(avg) remains constant across physiological and pathological states. This assumption has not been consistently validated [151,152]. Despite this limitation, Chen’s method remains the most widely accepted noninvasive approach for Ees estimation [153]. However, its implementation requires specialized software and is not yet widely available for routine clinical use [138]. Moreover, for the RV, noninvasive single-beat estimation of Ees remains insufficiently validated, mainly due to the greater variability of RV pressure–time dynamics and geometric assumptions [154,155]. In both ventricles, the volumetric parameters required for these calculations are most accurately obtained from 3D echocardiography, which provides a more reliable representation of ESV and EDV compared with conventional 2D imaging [11,156]. Because both Ees and Ea share the same Pes term, their ratio can be approximated noninvasively by SV/ESV, providing a simple surrogate of VA coupling [139,155,157]. Nevertheless, the TAPSE/pulmonary artery systolic pressure (TAPSE/PASP) ratio has emerged as a simple non-invasive surrogate of RV–PA coupling with established prognostic value [14,158,159].

Theoretical and experimental analyses have shown that maximum ventricular efficiency occurs when Ea is approximately half of Ees (Ea/Ees ≈ 0.5), while stroke work (SW) is maximized when Ea equals Ees (Ea/Ees ≈ 1.0). Therefore, VA uncoupling occurs when Ea exceeds Ees, expressed as an Ea/Ees ratio greater than 1, or equivalently as an Ees/Ea ratio below 1, resulting in reduced efficiency and impaired contractile performance [40,125,132,139]. For consistency, although LV–arterial coupling is most often described as the Ea/Ees ratio, RV–PA coupling is conventionally expressed as its inverse, Ees/Ea. Higher values of Ees/Ea indicate better coupling, whereas lower values reflect progressive uncoupling [49]. In physiological conditions, optimal RV–PA coupling is observed at Ees/Ea values between approximately 1.5 and 2.0. Uncoupling is generally defined when Ees/Ea falls below 1.0, indicating an inadequate contractile response to the prevailing afterload [154]. Importantly, in the right ventricle, a decline in the Ees/Ea ratio occurs earlier than any measurable reduction in intrinsic contractility or increase in EDV. This early decrease in Ees/Ea represents the initial stage of RV–PA uncoupling, preceding overt RV dilation and systolic failure, and therefore serves as a sensitive prognostic marker of impending right ventricular decompensation. From a clinical standpoint, this observation carries major therapeutic implications, as it identifies the critical point at which initiating targeted vasodilator or pulmonary vasomodulatory therapy may preserve RV–PA coupling and prevent further functional deterioration [4].

### 4.3. Cardiac Power Output

The CPO represents the total hydraulic energy that the heart delivers to the circulation per unit of time. It is regarded as an integrated measure of overall left ventricular pump performance because it reflects both the flow generated by the ventricle and the pressure against which it ejects blood. Mathematically, it is obtained by multiplying the mean arterial pressure by the cardiac output and a conversion factor (0.0022) to express the result in Watts. When normalized to body surface area, it yields the Cardiac Power Index (CPI), with normal resting values ranging between 0.5–0.7 W/m^2^, whereas normalization to LV mass (CPO/mass) provides an estimate of myocardial power generation per gram of tissue [160]. SW, defined as the external mechanical work performed by the ventricle during a single cardiac cycle, expresses work per beat, while CPO reflects work per minute. These two parameters are strongly correlated, since both describe the mechanical energy output of the left ventricle [161,162]. In contrast, LVEF shows only a weak or inconsistent correlation with SW or indices of cardiac power, since it is mainly a volumetric measure influenced by loading conditions [163]. The maximal cardiac pumping capability can be defined by the maximum CPO (CPOmax) achieved during maximal physiological stimulation or exercise, while the difference between resting and peak values defines the cardiac power reserve, an important index of functional and contractile reserve. Recent studies have demonstrated that CPOmax is a strong determinant of exercise capacity and one of the most powerful prognostic predictors in both chronic and acute HF [160]. In clinical practice, CPO can be estimated non-invasively using 2D or 3D-derived CO together with cuff-measured arterial pressure, both at rest and during exercise or stress conditions to determine CPOmax. CO can be calculated as LV outflow tract (LVOT) area multiplied by the LVOT velocity–time integral (VTI) and heart rate using 2D echocardiography, or alternatively from 3D-derived stroke volume multiplied by heart rate, or by combining a 3D-derived LVOT area with LVOT VTI measurements [161,164].

### 4.4. Speckle Tracking Echocardiography

2D STE is an advanced imaging technique used for the quantitative assessment of myocardial deformation. Deformation refers to the change in myocardial shape or dimension during the cardiac cycle [165]. Strain, also known as Lagrangian strain, represents the change in myocardial fiber length at the end of contraction compared with its original unstressed length. It is a dimensionless parameter, expressed as a percentage, and reflects myocardial shortening, thickening, or lengthening depending on fiber orientation [166,167]. Similar to TDI, STE provides information on myocardial velocity and deformation indices such as strain and strain rate, which help evaluate systolic and diastolic function, myocardial mechanics, and myocardial ischemia. 2D STE analyzes standard B-mode images using specialized post-processing software that tracks natural acoustic markers called “speckles” within the myocardium [168]. These are created by random scattering of ultrasound waves. The speckles form stable acoustic patterns within the LV wall that can be followed frame by frame throughout the cardiac cycle. In practice, the software tracks small clusters of speckles, known as kernels, as natural acoustic markers of myocardial motion, allowing objective quantification of both regional and global deformation [166]. Three main orthogonal components of strain are recognized, longitudinal, circumferential, and radial. It is important to note that, because myocardial fibers have different orientations, contraction is a complex 3D process. Clinically, only the three main orthogonal strain directions are assessed. However, six additional shear components also exist in 3D space, the most relevant being the circumferential–longitudinal LV shear strain, which represents rotation, twist, and torsion [168,169]. Among deformation indices, global longitudinal strain (GLS) is the most widely adopted metric. It predominantly reflects subendocardial longitudinal fibers which are highly susceptible to early ischemia and wall stress [170]. Moreover, GLS shows lower inter- and intraobserver as well as intervendor variability compared with circumferential, radial strain, and torsion, providing robust reproducibility and clinical applicability [171,172,173,174]. GLS normal ranges are closely related to age, sex, and vendor. In healthy individuals, LV GLS is approximately −20%, with reported normal ranges between −18% and −22%, while values less negative than −16% are generally considered abnormal [175,176]. Practical acquisition considerations for GLS imaging are summarized in Table 2.

**Figure 4:**
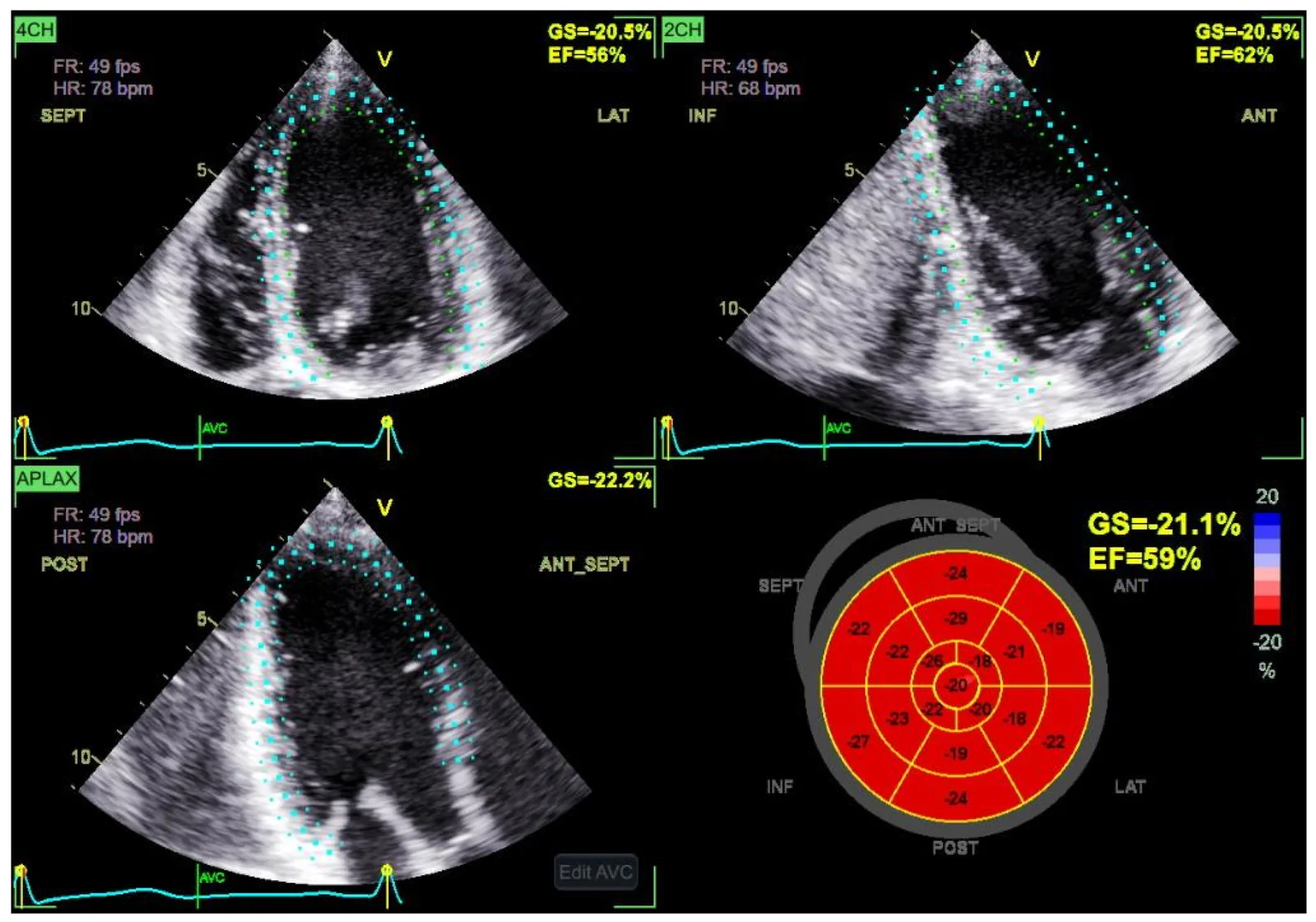
GLS assessment of the left ventricle, showing the three apical views, 4-chamber, 2-chamber, and 3-chamber, and the corresponding bull’s-eye strain map. Images were acquired during sinus rhythm at a frame rate of 49 frames/s.

Myocardial strain and its sensitive index of myocardial deformation, GLS, provides a widely accepted, accurate and repeatable evaluation of LV systolic function with STE as the main tool for their assessment [177]. The subendocardial layer predominantly contributes to longitudinal shortening, the mid-wall layer to radial thickening through circumferential shortening, and the subepicardial layer to circumferential and rotational mechanics responsible for LV twist [19]. There are several conditions in which the EF remains largely unaffected, whereas the subendocardial layer is significantly impaired [178,179,180]. While both strain and LVEF measure LV performance, there is a fundamental difference between the two. Myocardial strain quantifies myocardial deformation and provides a sensitive marker of LV systolic function, whereas LVEF represents a volumetric index of ventricular chamber performance, derived from the ratio of SV to EDV. Even when contractility is reduced, compensatory mechanisms such as ventricular dilation or geometric adaptation may preserve a normal SV at rest [106]. Therefore, STE is particularly suited for evaluating global and regional systolic function in patients with HF and apparently normal EF [181]. Regional dysfunction may not be detected when relying solely on a global index like LVEF. Moreover, accurate LVEF calculation relies on geometric assumptions and is affected by intra- and interobserver variability, all of which can introduce significant measurement error. In addition, LVEF is much more load-dependent than strain [182]. In contrast, myocardial strain reflects deformation at the myocardial level and can uncover subtle abnormalities in contractility on a more fundamental level, in an early stage of disease. Although longitudinal strain and EF correlate well in healthy subjects, this relationship becomes weaker in conditions such as myocardial infarction or HF, confirming that the two parameters describe distinct aspects of LV systolic function. Hence, STE offers complementary and clinically meaningful information beyond traditional LVEF assessment [168] (Figure 4).

**Figure 5:**
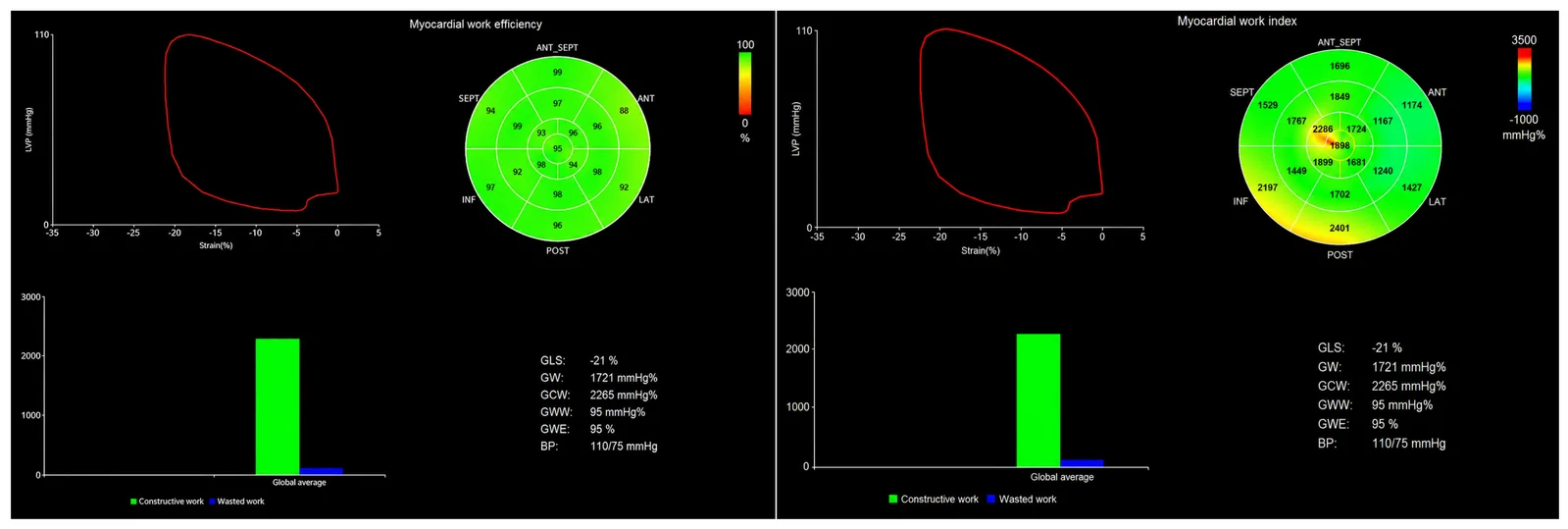
Myocardial work assessment derived from non-invasive left ventricular pressure–strain loop analysis after incorporating brachial cuff blood pressure, illustrating global work index (GWI), global constructive work (GCW), global wasted work (GWW), and myocardial work efficiency (MWE), displayed as corresponding bull’s-eye maps.

MW further refines the assessment of LV systolic performance, providing complementary information to GLS, by integrating myocardial deformation with afterload through non-invasive pressure–strain loops. In physics, work is the product of force and distance. In the heart, this corresponds to the pressure it generates multiplied by the volume of blood ejected during each beat [183]. The classic invasive representation of this relationship is the PV loop, where the area enclosed equals stroke work which is the mechanical energy the left ventricle expends each cycle. Although PV loops provide valuable mechanistic insight, their acquisition is invasive and time-consuming. MW offers a non-invasive alternative by using brachial cuff systolic pressure to scale an estimated LV pressure curve and synchronizing it with speckle-tracking strain and valve-event timing [184,185]. Because MW reflects the mechanical energy generated by the myocardium during contraction, it correlates closely with myocardial oxygen consumption, providing insight into both contractile performance and energetic efficiency [184]. This approach quantifies both total systolic work, expressed as the Global Work Index (GWI), and its partition into constructive and wasted components, expressed as Global Constructive Work (GCW) and Global Wasted Work (GWW), respectively, yielding a measure of efficiency known as Myocardial Work Efficiency (MWE). All MW parameters are expressed in mmHg%, except for MWE, which is expressed as a percentage (%). In healthy individuals, normal MWE values are approximately 95%, while reference ranges and cut-offs for GWI, GCW, and GWW have been reported by population-based studies such as EACVI NORRE, STAAB, Galli et al., and the Copenhagen City Heart Study, according to age and sex [186,187,188,189]. By incorporating conditions into its analysis, MW helps differentiate true contractile impairment from afterload-related changes in strain, enhancing the accuracy of systolic function assessment. MW can clarify whether a decrease in GLS reflects intrinsic myocardial dysfunction or merely the hemodynamic burden imposed by elevated afterload [190] (Figure 5).

It’s important to note that MW depends on accurate brachial cuff pressure and appropriate timing of valvular events [184]. Even under physiological conditions, LV systolic pressure is estimated from brachial systolic pressure rather than directly measured, which remains an inherent assumption of the method [191]. Its reliability may be reduced when brachial cuff pressure deviates substantially from true LV ESP, such as in cases of marked pressure amplification, significant aortic valve stenosis, LVOT obstruction, or severe MR, as well as in the presence of mechanical dyssynchrony, such as left bundle branch block (LBBB) [192]. It should also be acknowledged that MW is currently supported only by General Electric (GE) machines, which may limit its wider clinical implementation [193].

**Figure 6:**
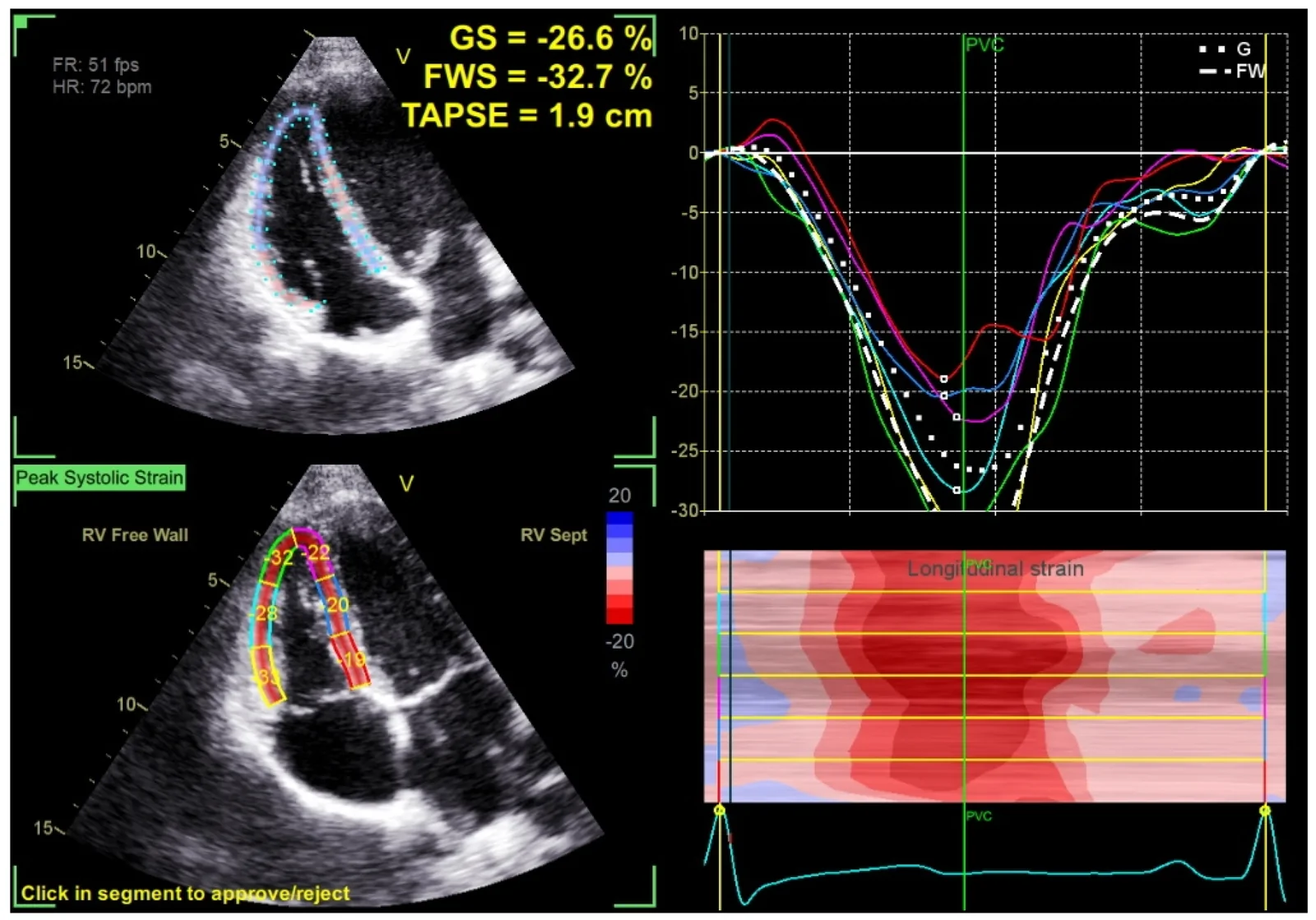
Right ventricular longitudinal strain analysis, demonstrating right ventricular global longitudinal strain (RV GLS; displayed as GS by the software) and free-wall longitudinal strain (FWS). Analysis was performed from a right ventricular-focused apical four-chamber view acquired during sinus rhythm at a frame rate of 51 frames/s. All six right ventricular segments are displayed, and the corresponding strain curves illustrate regional longitudinal deformation throughout the cardiac cycle.

Assessment of RV function has long been challenging due to its complex geometry and anatomy. The introduction of STE has allowed for a more objective and quantitative evaluation of RV systolic performance, complementing conventional echocardiographic parameters. Compared with the left ventricle, RV longitudinal strain by 2D STE is derived exclusively from the apical four-chamber view and can be calculated either as the average of the septal and free-wall GLS or as free-wall longitudinal strain (FWS) alone [194]. Although reference values may vary according to methodological and population-related factors, recent meta-analyses suggest that normal RV FWS is approximately −27%. According to the latest ASE/EACVI consensus statement, the lower limit of normal for RV FWS is −20% in men and −21% in women, with less negative values considered abnormal [176,195,196]. 2D RV longitudinal strain has emerged as a promising method for assessing RV performance in a wide range of cardiac conditions. Despite its growing clinical use, RV longitudinal strain analysis presents several technical and methodological challenges. The currently available software was originally designed for the left ventricle and later adapted for the right ventricle, whose irregular geometry and thinner wall make tracking more complex. The RV inflow and outflow tracts lie in different imaging planes, and the thin free wall makes it difficult to separate the myocardium from the pericardium, especially when image quality is suboptimal. Because the imaging orientation of the right ventricle is reversed compared with the left ventricle, the conventional bull’s-eye plots, polar maps designed for LV segmental deformation, are not directly applicable, making the visualization and interpretation of regional systolic function less straightforward. These factors reduce reproducibility and increase inter-observer variability [194,195]. Therefore, accurate RV FWS analysis requires high-quality imaging and experienced operators. Nevertheless, RV FWS remains a valuable and increasingly utilized parameter for the early detection of subtle RV systolic dysfunction (Figure 6).

Practical acquisition considerations for RV longitudinal strain imaging are summarized in Table 2.

STE offers several advantages but also certain technical limitations that should be considered when interpreting its results. Compared with cardiac MRI, it is more widely available, less expensive, and can be performed at the bedside with shorter acquisition and processing times. Unlike TDI, STE is independent of the insonation angle, as speckles can be tracked in any direction within a 2D or 3D dataset. It also does not require very high frame rates and allows simultaneous evaluation of longitudinal, circumferential, and radial strain. In practice, optimal frame rates are above 30 Hz, ideally around 50 Hz, to ensure accurate tracking [167]. Another important advantage is the elimination of the “tethering effect” seen with TDI, where non-contractile segments are passively pulled by adjacent viable myocardium, mimicking active contraction. Since STE derives strain from speckle motion rather than tissue velocities, this artifact is avoided [168]. Despite these benefits, 2D STE remains technically demanding. Image quality is crucial, and some myocardial segments, particularly apical ones, may be inadequately visualized [197]. Out-of-plane motion can reduce accuracy, although emerging 3D STE may help address this issue [168]. Differences in vendor-specific software algorithms also lead to variability in strain values, limiting inter-vendor comparability [198]. Accurate delineation of endocardial and epicardial borders remains difficult, often requiring manual adjustment. Most software assumes uniform myocardial thickness, which does not always reflect true anatomy, and variations in ventricular size and shape may hinder full visualization, especially in dilated hearts [168]. Despite these limitations, STE remains a robust and reproducible method that provides useful insights into myocardial mechanics and systolic performance.

3D STE represents an emerging development in myocardial deformation imaging, providing a more comprehensive depiction of cardiac motion. Conventional 2D STE evaluates longitudinal, circumferential, and radial strain, but this remains a simplification of the complex myocardial fiber contraction pattern, as it neglects shear strains and out-of-plane motion. By capturing deformation throughout the cardiac volume, 3D STE allows simultaneous quantification of strain components in all directions, providing a more realistic understanding of myocardial mechanics [199,200,201,202]. Preliminary studies have shown that 3D STE can assess both left and right ventricular function, including indices such as longitudinal, circumferential, radial, and area strain. However, its use, particularly for RV analysis, remains at an early stage. The lack of standardized reference values, acquisition protocols, and inter-vendor reproducibility currently limits its clinical adoption. Despite encouraging data, several technical and practical challenges persist. High image quality, regular cardiac rhythm, and advanced post-processing are essential, while relatively low temporal resolution, high cost, and vendor variability further constrain its widespread use [203,204]. Consequently, although 3D STE provides valuable information on complex myocardial deformation, it currently remains primarily a research tool pending further validation in larger studies [168,194].

### 4.5. Therapeutic Implications

Longitudinal strain (LS) integrates both segmental and global systolic performance. Several conditions may exhibit preserved EF despite substantial subendocardial dysfunction. This pattern is classically seen in arterial hypertension, where early subendocardial impairment precedes overt systolic failure [178]. Furthermore, reduced strain in the affected segments has been demonstrated in both acute ischemia and myocarditis, preceding any measurable decline in EF and providing valuable diagnostic and prognostic insights [179,180]. Importantly, in patients with suspected coronary artery disease, resting GLS has demonstrated diagnostic accuracy comparable to stress echocardiography variables for the detection of obstructive coronary disease [205]. In hypertrophic cardiomyopathy, GLS is selectively reduced whereas circumferential and radial strain, as well as LVEF, remain preserved. STE distinguishes forms of LV hypertrophy, with LS more reduced and heterogeneous in hypertrophic cardiomyopathy than in hypertensive or athletic hypertrophy [206]. Of note, a reduction in GLS reclassifies a substantial proportion of patients from Stage A to Stage B HF, while in Stages C and D heart failure with reduced ejection fraction (HFrEF), worsening GLS provides superior prognostic discrimination compared with LVEF [207,208]. In acute heart failure, GLS shows strong associations with both short- and long-term outcomes compared with LVEF [209]. Furthermore, in cardio-oncology, GLS enables risk stratification of high-risk patients and guides the initiation of cardioprotective therapy, even in the absence of an overt decline in LVEF [207]. Additionally, characteristic strain patterns may assist in differentiating among various pathological conditions [180].

Recent data further highlight the prognostic value of deformation imaging beyond standard volumetric indices. In ischemic HF patients undergoing cardiac resynchronization therapy (CRT), impaired baseline GLS together with elevated N-terminal pro–B-type natriuretic peptide (NT-proBNP) independently predict adverse long-term outcomes [177]. Moreover, the presence of mechanical dyssynchrony, particularly a septal-to-posterior wall delay ≥130 ms on speckle-tracking radial strain, strongly predicts both immediate hemodynamic improvement and long-term reverse remodeling. In contrast, its absence is closely associated with non-response to CRT [210]. Additionally, in severe aortic valve stenosis with preserved EF, a GLS value around −15% identifies patients with concealed myocardial damage and a higher risk of deterioration. GLS-based staging reclassifies many patients into higher-risk stages, revealing more extensive myocardial and extracardiac damage associated with higher mortality. Therefore, GLS refines risk stratification beyond standard echocardiography and biomarkers. This, in turn, may help guide earlier intervention in high-risk patients with severe aortic valve stenosis despite preserved LVEF [211]. In conclusion, STE provides incremental diagnostic and prognostic value beyond conventional indices, allowing more informed clinical and therapeutic decision-making.

CPO has emerged as a superior index of global left ventricular performance compared with LVEF. Its prognostic value has been consistently demonstrated across the spectrum of HF. In advanced HF, a CPI below 0.44 W/m^2^ was associated with a 2.4-fold higher risk of death, transplantation, or LVAD implantation [212]. Similarly, peak cardiac power ≤ 1.5 W during maximal bicycle exercise identified patients with poor outcomes [213]. In patients with preserved EF, stress-derived CPO normalized to LV mass independently predicted mortality and new-onset HF, while higher values indicated favorable prognosis [161]. Importantly, CPO has been shown to be the strongest independent haemodynamic predictor of mortality in cardiogenic shock, surpassing traditional invasive parameters [214]. Collectively, these findings highlight the clinical importance of CPO as a therapeutic and prognostic parameter that more accurately reflects the heart’s true pumping capability than conventional indices such as LVEF [215].

In pulmonary arterial hypertension (PAH), RV function is a major determinant of clinical outcomes, as patient survival is largely dependent on the ability of the right ventricle to adapt to increased afterload [49]. Echocardiographic parameters reflecting RV size and systolic function have been shown to correlate with disease severity and prognosis in patients with PAH [216]. STE, particularly RV GLS, allows the detection of early systolic dysfunction and has been associated with adverse outcomes in PAH, often providing incremental prognostic information over conventional echocardiographic indices [217,218,219]. Early identification of RV dysfunction may therefore have important therapeutic implications, enabling timely treatment escalation, closer follow-up, and referral for advanced therapies before the development of RV-PA uncoupling.

### 4.6. Gaps in Evidence and Future Perspectives

LVEF, despite its widespread clinical use, is a geometric ratio that is neither a true measure of intrinsic myocardial contractility nor an accurate representation of actual pump performance, and it fails to reliably discriminate prognosis across the HF spectrum [163,215]. GLS is a sensitive early marker of myocardial dysfunction, yet its true independence from loading conditions remains uncertain. Emerging approaches that integrate preload and afterload, such as the recently proposed integrated myocardial work index (iMWI), may help overcome this limitation, but require prospective validation [220]. Additional load-integrated energetic indices, such as Global Longitudinal Active Strain Energy Density (GLASED), which incorporate myocardial deformation, arterial pressure, ventricular geometry and wall stress into a unified load-adjusted measure of contractile performance, also represent a promising direction but remain at an early research stage [221]. Furthermore, current RV strain algorithms are adapted from LV-based software, a limitation that underscores the need for RV-specific models to improve accuracy and reproducibility [194]. RV MW is also emerging as a promising load-adjusted approach, although further validation and standardization are required before its routine clinical use [222]. 3D echocardiography improves the accuracy of cardiac chamber volumes to a level comparable with CMR [114,120]. It also enables more advanced STE analysis. However, 3D STE remains primarily a research tool pending reproducibility and outcome data from large multicenter studies [168,194]. CPO, a robust marker of global pump performance with established prognostic value across the HF spectrum and in cardiogenic shock, is still underutilized in routine practice [160,214,215]. Future work should focus on integrating load-adjusted deformation indices, validating 3D STE, and defining the clinical utility of global pump metrics to refine risk stratification and guide management.

## 5. Conclusions

Estimating the intrinsic contractile function of both ventricles remains challenging in clinical practice. Although classical echocardiographic indices such as LVEF, lateral and septal mitral S′, tricuspid S′, FAC, and TAPSE are widely used, their inability to accurately reflect intrinsic myocardial contraction limits their diagnostic and prognostic utility in patients with true systolic dysfunction.

Incorporating novel echocardiographic parameters, including speckle-tracking strain of both ventricles, 3D volumetric assessment, MW, and CPO, into routine evaluation, alongside conventional echocardiographic indices, has the potential to provide more accurate, reproducible, and clinically meaningful insights. Although still underutilized, these advanced indices may ultimately allow a more objective characterization of HF and a more precise estimation of patient prognosis.

## Figures and Tables

**Table 1:** Conventional and novel echocardiographic indices of left and right ventricular systolic function.

Variable	Indices	Challenges	Indices with Prognostic Value
**Conventional indices of LV systolic function**	(1) LVEF	Regional WMAs Geometrical assumptions Foreshortened views Endocardial delineation Load-dependent	Non-specific prognostic marker
	(2) eFS & FSmw	Regional WMAs Geometrical assumptions Foreshortened views Endocardial delineation Load-dependent Persistent preload dependence of FSmw Non-integrated, complex FSmw calculations	Limited prognostic evidence
	(3) dP/dt from the MR jet	Load-dependent Requires a well-defined MR jet	Limited prognostic evidence
	(4) Septal S’ & lateral S’	Load-dependent Angle-dependent Limited global index Mitral annulus calcification MV repair or replacement Tethering effect	Established prognostic value
**Conventional indices of RV systolic function**	(1) TAPSE & Tricuspid S’	Load-dependent Angle-dependent Limited global index Tethering effect Severe TR Tricuspid annulus calcification TV repair or replacement	Established prognostic value
	(2) dP/dt from the TR jet	Load dependence Requires a well-defined TR jet Lack of robust normative data	Limited prognostic evidence
	(3) RIMP	Load-dependent Difficulty in IVCT/IVRT delineation Modest intermethod correlation	Established prognostic value
	(4) FAC	Manual tracing of the RV endocardial edge	Established prognostic value
	(5) 2D RVEF	Not guideline-supported	Not established
**Novel indices**	(1) 3D LVEF & 3D RVEF	Load-dependent Automated endocardial detection may reduce accuracy Reference values not well defined	Established prognostic value
	(2) TAPSE/PASP ratio	TR-dependent Load-dependent PASP estimation variability	Established prognostic value
	(3) 2D LV GLS	Load-dependent Image quality-dependent Out-of-plane motion Inter-vendor variability	Established prognostic value
	(4) RV FWS	Load-dependent Complex geometry Thin wall-related tracking difficulty Inter-observer variability Inter-vendor variability	Established prognostic value
	(5) Non-invasive MW	No universally established cut-off values Preload dependence Less afterload-dependent than GLS Limited validity in abnormal pressure–strain sequences	Established prognostic value
	(6) 3D STE	Remains primarily a research tool	Under investigation
	(7) iMWI	Limited validation data	Under investigation
	(8) CPO	Accurate (often invasive) blood pressure	Established prognostic value

CPO, cardiac power output; EF, ejection fraction; eFS, endocardial fractional shortening; FAC, fractional area change; FSmw, fractional shortening mid-wall; GLS, global longitudinal strain; iMWI, integrated myocardial work index; IVCT, isovolumic contraction time; IVRT, isovolumic relaxation time; LVEF, left ventricular ejection fraction; MR, mitral regurgitation; MV, mitral valve; MW, myocardial work; PASP, pulmonary artery systolic pressure; RIMP, right ventricular index of myocardial performance; RV, right ventricular; RV FWS, right ventricular free-wall strain; RVEF, right ventricular ejection fraction; S’, systolic velocity; STE, speckle tracking echocardiography; TAPSE, tricuspid annular plane systolic excursion; TR, tricuspid regurgitation; TV, tricuspid valve; WMAs, wall motion abnormalities.

**Table 2:** Practical Guidance for Measurement Using 3D Echocardiography and Speckle Tracking Echocardiography.

Imaging Modality	Practical Acquisition Recommendations
General Longitudinal Strain Principles	Stable ECG gating and clear endocardial borders. Measured from representative beats and averaged over multiple cycles in irregular rhythm, excluding ectopic beats. Frame rate > 30 Hz (recommended 40–80 frames/s), optimized by narrowing sector width and reducing imaging depth. Manual ROI adjustment should be performed when necessary to ensure optimal myocardial tracking prior to accepting GLS measurements. Maintain similar loading conditions in follow-up studies. Do not compare strain values across vendors/software platforms.
2D LV GLS	Acquire apical 4-, 2-, and 3-chamber views avoiding foreshortening. Do not report GLS if ≥2 segments show inadequate tracking or if one apical view is non-diagnostic.
RV FWS & RV GLS	Use RV-focused apical 4-chamber view. Report RV FWS (3 free-wall segments) and/or RV GLS (6 segments: 3 free-wall + 3 septal segments). Carefully adjust the ROI to include the thin RV myocardium while excluding pericardium and trabeculations.
3D Ventricular Volume Quantification	Prefer multi-beat acquisition in stable rhythm. Use single-beat acquisition in atrial fibrillation or irregular rhythm to avoid stitching artifacts. Inspect for stitching artifacts before analysis. Optimize temporal resolution by reducing sector width and imaging depth to include only the region of interest, targeting volume rates ≥ 20–25 volumes/s.

ECG, Electrocardiogram; FWS, Free-Wall Strain; GLS, Global Longitudinal Strain; LV, Left Ventricle; ROI, Region of Interest; RV, Right Ventricle.

## Data Availability

No new data were created or analyzed in this study. Data sharing is not applicable to this article.

## Abbreviations

CMR	cardiac magnetic resonance
CO	cardiac output
CPI	cardiac power index
CPO	cardiac power output
CRT	cardiac resynchronization therapy
Ea	arterial elastance
EDP	end-diastolic pressure
EDV	end-diastolic volume
EDPVR	end-diastolic pressure–volume relationship
eFS	endocardial fractional shortening
Ees	end-systolic elastance
ESP	end-systolic pressure
ESPVR	end-systolic pressure–volume relationship
ESV	end-systolic volume
EF	ejection fraction
FAC	fractional area change
FS	fractional shortening
GCW	global constructive work
GLASED	global longitudinal active strain energy density
GLS	global longitudinal strain
GS	global strain
GWW	global wasted work
GWI	global work index
HF	heart failure
HFrEF	heart failure with reduced ejection fraction
iMWI	integrated myocardial work index
IVC	isovolumic contraction
IVCT	isovolumic contraction time
IVRT	isovolumic relaxation time
LVEF	left ventricular ejection fraction
LVH	left ventricular hypertrophy
LVOT	left ventricular outflow tract
LS	longitudinal strain
MAP	mean arterial pressure
FSmw	midwall fractional shortening
MR	mitral regurgitation
MV	mitral valve
MW	myocardial work
MWE	myocardial work efficiency
NT-proBNP	N-terminal pro–B-type natriuretic peptide
PA	pulmonary artery
PAH	pulmonary arterial hypertension
PASP	pulmonary artery systolic pressure
PV	loop pressure–volume loop
RIMP	right ventricular index of myocardial performance
RVEF	right ventricular ejection fraction
STE	speckle-tracking echocardiography
SV	stroke volume
SW	stroke work
S′	systolic velocity
TDI	tissue Doppler imaging
TAPSE	tricuspid annular plane systolic excursion
TR	tricuspid regurgitation
TV	tricuspid valve
VA	coupling ventriculo–arterial coupling
VTI	velocity time integral
WMAs	wall motion abnormalities
